# Oral health knowledge, attitudes, and practices and oral health-related quality of life among stroke inpatients: a cross-sectional study

**DOI:** 10.1186/s12903-022-02446-1

**Published:** 2022-09-19

**Authors:** Simin Huang, Yangyang Liu, Muling Li, Zhihong Liu, Fang Zhao, Jinjun Li, Huiqi Lu, Hongzhen Zhou

**Affiliations:** 1 Department of Nursing, Nanfang Hospital, Southern Medical University, No.1838 Guangzhou Avenue North, Guangzhou, 510515 China; 2 School of Nursing, Southern Medical University, Guangzhou, China; 3 Nanfang Hospital, Southern Medical University, Guangzhou, China; 4 Department of Neurology, Guangdong 999 Brain Hospital, Guangzhou, China; 5 Department of Stomatology, Nanfang Hospital, Southern Medical University, Guangzhou, China

**Keywords:** Stroke, Oral health, Knowledge, Attitudes, Practices, Oral health-related quality of life

## Abstract

**Background:**

Stroke patients have poor oral hygiene, experience oral dysfunction due to disease factors, and have impaired oral health-related quality of life (OHRQoL). This study aimed to determine the oral health knowledge, attitudes, and practices of stroke inpatients, assess the OHRQoL of these patients, and identify their correlates.

**Methods:**

In this cross-sectional study, 281 stroke inpatients aged between 22 and 88 years (57.94 ± 10.94) were conveniently selected from three hospitals in Guangzhou, China. OHRQoL was measured among these stroke patients using a Chinese version of the Oral Health Impact Profile-14 (OHIP-14). SPSS 26.0 was used for statistical analysis. Mean scores, standard deviations, and frequency distributions were obtained. The Mann–Whitney *U* test, Kruskal‒Wallis *H* test, Spearman's correlation, and multiple linear regression were used in the analysis.

**Results:**

The mean score of the patients' OHRQoL was 8.37 ± 6.67, with the highest score in the pain or discomfort of the mouth dimension (3.11 ± 2.13) and pain being the most common negative effect (13.5%). In multiple linear regression analysis, significant differences were found between patients only in age (*P* = 0.008), toothache (*P* < 0.001), self-rated oral health (*P* < 0.001), time since last dentist visit (*P* = 0.037) and reason for not having visited a dentist in the past year (*P* < 0.001).

**Conclusion:**

The OHRQoL of patients hospitalised with stroke was moderate, and oral conditions still need to be improved. Increasing age, toothache, a longer time since the last dental visit and the reason for not visiting a dentist in the past year had a negative effect on OHRQoL, and better self-rated oral health had a positive effect. Therefore, in clinical work, greater attention should be given to elderly stroke patients, patients with poor oral status and poor oral health behaviours, timely assessment of patients’ swallowing function, nutritional function, and self-care ability, and early and targeted oral health interventions and guidance.

## Background

Worldwide, stroke is a very common cause of long-term disability, morbidity, and mortality. Oral hygiene after stroke is often neglected due to neurological deficits, physical weakness, lack of coordination, cognitive dysfunction, and prioritization of other health needs [1]. However, oral dysfunction is highly prevalent after stroke and includes difficulty swallowing and eating, both of which may affect nearly 80% of poststroke patients [2]. Patients exhibit mastication, swallowing, and speech [2] impairments, which can be incredibly disabling and are often not fully reversible [3]. Stroke affects not only oral sensory and orofacial function but also oral hygiene [4]. In addition, medications used in stroke treatment can further compromise oral hygiene status as they reduce salivary flow [5]. The rapid multiplication of oral pathogens can lead to the mouth becoming a reservoir of pathogens [6]. A meta-analysis found that stroke patients had greater tooth loss, more dental caries, and more severe periodontal disease than nonstroke controls [7]. It is well established that patients with a history of stroke have much poorer oral hygiene than healthy older adults without a history of stroke [8]. This complication from a lack of oral hygiene care can impede stroke recovery, prolong hospital stays and potentially increase mortality [9].

With the shift in the biopsychosocial medicine paradigm, the construct of oral health-related quality of life (OHRQoL) as a subset of overall health-related quality of life has been established and widely recognized over the past decades [10–12]. Thus, OHRQoL reflects the potential impact of oral conditions, including dental, periodontal, and functional diseases, tooth loss, and various other pathologies on quality of life [11–13]. Currently, one of the most widely used tools to measure OHRQoL is the Oral Health Impact Profile (OHIP) and its shortened version (OHIP-14).

However, there are no reports on the level of knowledge, attitudes, and practices (KAP) regarding oral health among stroke patients in China, and there are fewer studies on OHRQoL among stroke patients. Therefore, this study aimed to determine a. the level of oral health KAP among stroke inpatients in general and cerebrovascular specialty hospitals in China and b. the factors influencing OHRQoL among stroke patients to provide a reference for oral health care for stroke patients.

## Methods

### Design and objective

This cross-sectional study was carried out in the neurology departments of two tertiary public hospitals and one tertiary private hospital between Nov 2021 and Feb 2022 in the urban area of Guangzhou, Guangdong Province, China. A convenience sampling method was used. The aims of this study were (1) to determine the current status of participants' oral health knowledge, attitudes, and behaviours and (2) determine the factors influencing the participants’ OHRQoL.

### Participants

The study population included stroke inpatients in the Department of Neurology, Nanfang Hospital of Southern Medical University, Baiyun Branch of Nanfang Hospital of Southern Medical University, and Guangdong 999 Brain Hospital. A total of 281 hospitalised stroke patients (219 males and 62 females) participated in this study. The inclusion criteria for this study were as follows: (1) patients who met the diagnostic criteria of “Diagnostic Points for Various Major Cerebrovascular Diseases in China 2019” and were diagnosed with stroke after computed tomography (CT) or magnetic resonance imaging (MRI) examination; (2) age ≥ 18 years; (3) inpatients who were conscious and had stable vital signs; (4) patients with the ability to read, communicate and understand; and (5) patients and caregivers who volunteered to participate in this study. The exclusion criteria were (1) patients with oral tumours or acute oral infections, difficulty in opening the mouth, or other oral trauma; (2) patients with other central nervous system diseases, malignant tumours, and other serious comorbidities or obvious complications; and (3) patients with tracheal intubation, tracheotomy, indwelling nasogastric tube, or nasogastric tube. The survey was conducted in the medical staff examination and assessment section by a nurse who was trained in neurological and dental expertise and skills and was proficient in the application of the swallowing function assessment, nutritional assessment and oral assessment tests.

### Ethical considerations

The purpose of this study was explained to the patient or caregiver by the investigator before the start of the study. All patients participated in the study voluntarily and had the right to withdraw from this study at any time and without interference with their treatment. Verbal and informed consent was obtained by the investigator from the patient or their caregiver before the relevant tests and assessments. Informed consent was signed by all participants in this study, and if the participant was a dependent, signed informed consent was obtained from their caregiver. The caregiver was the legal guardian, usually the patient’s spouse or adult child [14]. Participants were supervised while completing the questionnaire, which was returned immediately after completion. The Medical Ethics Committee of the Southern Hospital of Southern Medical University approved this study (NFEC-2022-015).

### Questionnaire design

The demographic information of the participants included 18 items, including sex, age, education level, occupation, monthly household income per capita, permanent address of the family, lifestyle and dietary habits, marital status, residence status, primary family caregiver after admission, number of strokes, type of stroke, time of the first stroke, chronic diseases, dentures, number of teeth, number of missing teeth and existing poor oral status. This questionnaire was based on select questions from the World Health Organization (WHO) *Oral Health Questionnaire for Adults* 5th edition [15] and the *Fourth National Oral Health Survey Questionnaire (Adult Version)* [16] in China. It was a three-part questionnaire to investigate the oral health KAP of patients hospitalised with stroke. In this study, the Cronbach′s α coefficient for the knowledge and attitudes section of this questionnaire was 0.81.

The first section was an oral health knowledge survey consisting of 8 questions answered correctly, incorrectly or “do not know”. The questions were as follows: (1) it is normal for gums to bleed when brushing; (2) bacteria can cause inflammation of the gums; (3) brushing is not useful in preventing bleeding gums; (4) bacteria can cause tooth decay; (5) eating sugar can cause tooth decay; (6) fluoride is not useful in protecting teeth; (7) brushing protects teeth; and 8) oral disease may affect the health of the entire body’. Finally, patients were asked about how they learned about oral health. The overall rate of oral health knowledge was equal to the total number of knowledge questions answered correctly/(number of knowledge items per questionnaire x the number of participants with valid answers) × 100%.

In the second section, attitudes towards oral health were ascertained through five questions. The responses were agree, disagree and do not know. The questions were as follows: (1) Oral health is important to one’s life; (2) regular oral check-ups are essential; (3) good or bad teeth are innate and have little to do with one’s own protection; (4) preventing dental diseases depends on oneself first and foremost; and (5) maintaining oral health promotes one’s own health. The total rate of positive attitudes towards oral health was equal to the total number of positive attitude questions/(the number of attitude items in each questionnaire x the number of participants with valid responses × 100%).

In the third section, oral health practices were examined through 12 questions: (1) frequency of brushing or rinsing (3 times a day, 2 times a day, once a day, 3–6 times a week, 1–2 times a week); (2) oral cleaning methods (toothbrush, electric toothbrush, floss, toothpick, mouthwash); (3) toothpaste used (fluoride, no fluoride, none or unknown); (4) mouthwash (tap water, warm water, physiological saline, chlorhexidine, other); (5) frequency of toothbrush replacement (3 months, 3–6 months, 6–12 months, 1–2 years, replace when broken); (6) primary oral cleaner after hospitalization (self, family, nurse, caregiver, none); (7) denture cleaning method (Questions 7–8 were skipped if no dentures. Boiling water soak, warm water soak, cold water soak, toothbrush soak, disinfectant soak, other); (8) frequency of denture cleaning (3 times a day, 2 times a day, once a day, 3–6 times a week, 1–2 times a week); (9) time since the last dentist visit (within 6 months, 6–12 months, 1–2 years, 2–5 years, more than 5 years, have not been or do not remember); (10) reason for the last dentist visit (have not been or do not remember, seeking advice or recommendations, tooth, gum or mouth pain and discomfort, treatment or follow-up, routine check-up or treatment, other); (11) reasons for not having visited a dentist in the past year (no dental problems, dental disease not serious, no time, financial difficulties, fear of epidemic transmission, difficulty in registering or no dentist nearby, fear of painful dental visits, other); and (12) frequency of dental cleaning in the clinic or hospital (every 3 months, every 6 months, every year, every 2 years, every 3 years or more, never).

### Instruments

#### Barthel index (BI)

The Barthel Index (BI) was first described in the 1950s, and it is an interview-based approach to assess participants’ activities of daily living (ADL) [17]. It consists of 10 items including feeding, bathing, grooming, dressing, bladder control, bowel control, toilet use, moving, transferring, and going up and down stairs. Scores range from 0 (fully independent) to 100 (fully independent) depending on the patient’s independence in each task [18]. Studies have shown that the BI has good reliability and is suitable for the assessment of poststroke patients [19].

### Nutritional risk screening (NRS 2002)

This screening tool developed by the Danish Society for Parenteral and Enteral Nutrition scores patients on two separate components, (1) undernutrition and (2) disease severity, depending on whether they are absent, mild, moderate, or severe, with a total score of 0–6. Patients achieving a total score of ≥ 3 are classified as having nutritional risk [20].

### Water-swallowing test (WST)

The Kubota drinking water test, which was proposed by the Japanese scholar Toshio Kubota, is graded and simple to perform [21] and is a sensitive screening tool that is widely used in neurology departments in China. The WST is usually performed with 90 ml of clear liquid, but the risk of aspiration, asphyxia, and other complications in patients in the acute phase of stroke cannot be ignored when large amounts of water are used in screening. Therefore, a modified version of the WST using a smaller amount of water (30 ml) was used in this study. The patient was asked to drink 30 ml of warm water from a cup while sitting in an upright position to observe the time required to drink and choking. Grade I meant that the patient swallowed the water smoothly in one sitting within 5 s, Grade II meant that the patient swallowed the water in more than 2 parts without choking, Grade III meant that the patient swallowed the water in one sitting with choking, Grade IV meant that the patient swallowed the water in more than 2 parts with choking, and Grade V meant that the patient choked frequently and could not swallow all the water. A grade I patient was considered normal; a grade I patient who swallowed the water in more than 5 s,a grade II patient who was suspected to have a swallowing disorder, and grade III patients and above were considered to have dysphagia.

### Self-rated oral health and general health

The self-rating of oral and physical health was assessed. A 5-point scale was used for assessment (1 = ”very poor”, 2 = ”poor”, 3 = ”fair”, 4 = ”good”, 5 = ”very good”) [22].

### Oral health impact profile-14 (OHIP-14)

The impact of OHRQoL was measured using the Chinese continental version of the Oral Health Impact Profile-14 (OHIP-14), validated by domestic scholars, with a Cronbach′s alpha coefficient of 0.93 in the Chinese version. Four common factors were extracted from the 14 entries: diminished independence, psychological discomfort, discomfort in physical functioning, and pain and discomfort of the mouth, with a cumulative contribution of 72.6% [23]. The questionnaire included 14 problems related to the experience: articulation difficulties, degradation of taste, pain, discomfort during eating, self-consciousness, emotional tension, dissatisfaction with eating, interruption of eating, difficulty relaxing, embarrassment, irritability, inability to complete daily tasks, reduced satisfaction with life, and complete inability to work. The frequency of occurrence was assessed on a five-point Likert scale: 0 = never, 1 = seldom, 2 = sometimes, 3 = frequently, and 4 = very often. All values were summed to calculate a total OHIP-14 score, which can vary between 0 and 56; the higher the OHIP-14 score, the worse the OHRQoL. The options “very often” or “often” were considered to have a negative impact on the patient. In the present study, the Cronbach′s α coefficient for the OHIP-14 was 0.87.

#### Statistical analysis

Data were analysed using IBM^®^ SPSS^®^ Statistics 26.0. Means ± standard deviations or frequencies and percentages were used to describe participants’ demographic information and oral health KAP and self-rated general and oral health status. The OHIP-14 score data were nonnormally distributed, and the Mann‒Whitney U test and Kruskal‒Wallis H test were uesed to assess differences in sample characteristics. Spearman’s correlation was used to assess the correlation between the variables and OHRQoL. All significant variables were entered into a multiple linear regression with OHRQoL as the dependent variable, and a stepwise regression method was performed to control for the effects of possible confounding factors. Two-tailed tests were used in all analyses, and the significance level was set at *P* < 0.05.

## Results

### Participants

A total of 281 respondents out of an included sample of 300 completed surveys (response rate of 93.67%), comprising 219 (77.9%) males and 61 (22.1%) females. The participants were aged between 22 and 88 years, with a mean age of 57.94 ± 10.94 years. The majority of participants reported having an elementary school education and below (33.8%) or a junior high school education (38.4%). The majority of the participants were unemployed (65.8%), had an average monthly household income of less than 3,000 CNY (Chinese yuan) (61.6%), and had a permanent family address in the township (51.6%). Most of them had smoking (57.3%) and strong tea (61.6%) habits. The majority of participants were married (96.8%), lived with their spouse (71.2%), and were cared for by family members after hospitalization (82.2%). Most of them had first-episode stroke (71.2%), mainly ischemic stroke (89.3%), and the first stroke had occurred within one month (58.4%). Chronic diseases were present in 82.2% of the participants, and the majority had hypertension (70.1%). The demographic characteristics of the participants are shown in Table 1.

**Table 1:** Relationship between the participants’ demographic characteristics and OHRQoL was assessed using Mann–Whitney *U* test and Kruskal‒Wallis H test (N = 281)

Variable		Measures	*P*-value*
Gender (%)	Male	219(77.9)	0.382
	Female	62(22.1)	
Age, N (%)	18–44 years	28(10.0)	0.005
	45–59 years	125(44.5)	
	60–74 years	112(39.9)	
	75–89 years	16(5.7)	
Education level, N (%)	Elementary school and below	95(33.8)	0.053
	Junior high school education	108(38.4)	
	High school and secondary school	55(19.6)	
	College and above	23(8.2)	
Occupation, N (%)	Employed	96(34.2)	0.007
	Unemployed	185(65.8)	
Monthly household income per capita, N (%)	≤ 3000	173(61.6)	0.063
	3000–5000	82(29.2)	
	≥ 5000	26(9.3)	
Permanent address of the family, N (%)	Township	145(51.6)	0.959
	County	43(15.3)	
	Downtown	93(33.1)	
Lifestyle and dietary habits, N (%)	Smoking Yes No	161(57.3) 120(42.7)	0.243
	Drinking alcohol Yes No	91(32.4) 190(67.6)	0.724
	Sweet food Yes No	118(42.0) 163(58.0)	0.123
	Carbonated drinks Yes No	44(15.7) 237(84.3)	0.476
	Strong tea Yes No	173(61.6) 108(38.4)	0.374
	Pickled products Yes No	76(27.0) 205(73.0)	0.806
Marital status, N (%)	Married	272(96.8)	0.724
	Single	9(3.2)	
Residence status,N (%)	Living alone	20(7.1)	0.672
	Living with spouse	200(71.2)	
	Living with parents or children	59(21.0)	
	Other	2(0.7)	
Primary caregiver after admission to hospital, N (%)	Family members	231(82.2)	0.283
	Nursing worker	11(3.9)	
	Self-care	39(13.9)	
Number of strokes, N (%)	First onset	200(71.2)	0.381
	Recurrence	81(28.8)	
Type of stroke, N (%)	Hemorrhagic stroke	27(9.6)	0.679
	Ischemic stroke	251(89.3)	
	Mixed stroke	3(1.1)	
Time of first stroke, N (%)	Within 1 month	164(58.4)	0.567
	1–3 months	22(7.8)	
	3–6 months	19(6.8)	
	6–12 months	10(3.6)	
	Over 1 year	66(23.5)	
Chronic disease, N (%)	Yes	231(82.2)	0.485
	No	50(17.8)	
	Hypertension Yes No	197(70.1) 84(29.9)	0.291
	Diabetes Yes No	98(34.9) 183(65.1)	0.742
	Heart disease Yes No	17(6.0) 264(94.0)	0.311
	Chronic obstructive pulmonary disease Yes No	1(0.4) 280(99.6)	0.143
	Cancer Yes No	1(0.4) 280(99.6)	0.174
	Other chronic diseases 1 2 4 No	49(17.4) 3(1.1) 1(0.4) 228(81.1)	0.613
ADL, N (%)	Severe dependence	14(5.0)	0.572
	Moderate dependence	25(8.9)	
	Mild dependence	123(43.8)	
	Full self-care	119(42.3)	
Nutrition, N (%)	0	93(33.1)	0.953
	1	166(59.1)	
	2	21(7.5)	
	3	1(0.4)	
Swallowing function, N (%)	Grade I	247(87.9)	0.468
	Grade II	31(11.0)	
	Grade III	1(0.4)	
	Grade IV	1(.04)	
	Grade V	1(0.4)	
Dentures, N (%)	No	188(66.9)	0.009
	Partial	84(29.9)	
	All	9(3.2)	
Number of teeth, N (%)	< 10	25(8.9)	< 0.001
	10–20	40(14.2)	
	> 20	216(76.9)	
Number of missing teeth, N (%)	0–4	219(77.9)	< 0.001
	5–8	30(10.7)	
	> 9	32(11.4)	
Existing poor oral status, N (%)	Dry mouth Yes No	208(74.0) 73(26.0)	0.066
	Toothache Yes No	243(86.5) 38(13.5)	< 0.001
	Gingival bleeding Yes No	260(92.5) 21(7.5)	0.471
	Tooth decay Yes No	168(59.8) 113(40.2)	0.060
	Oral odor Yes No	144(51.2) 137(48.8)	0.193
	No Yes No	222(79.0) 59(21.0)	0.018

### Oral health knowledge, attitudes and practices

The mean scores for participants’ oral health knowledge and attitudes were 3.81 ± 2.06 (0–8) and 3.95 ± 1.31 (0–5), respectively. The overall oral health knowledge of stroke inpatients was 47.6%. However, only a minority of participants were aware of the benefits of fluoride (9.6%) and fissure sealants (11.4%) for their teeth. The majority of participants reported that they did not know about oral health (53.7%) or learned about it mainly from the internet (21.0%). The overall rate of positive attitudes towards oral health was 79.1%, with the majority of participants having positive attitudes towards oral health. However, more than one-third of the participants did not have positive attitudes about regular oral check-ups and the need for their own oral protection (36.7% and 39.9%, respectively). Specific values are shown in Tables 2 and 3.

**Table 2:** Relationship between the participants’ knowledge, attitudes and self-rated oral health and general health characteristics and OHRQoL was assessed using Spearman correlation test (N = 281)

Variable (range of scores)	M ± SD	Spearman correlation (r)	*p*-value
Knowledg(0–8)	3.81 ± 2.06	.060	0.315
Attitudes(0–5)	3.95 ± 1.31	− .007	0.901
Self general health assessment(1–5)	3.07 ± 0.93	− .146	0.018
Self-rated oral health assessment(1–5)	2.99 ± 0.87	− .365	0.015

*OHRQoL* Oral health-related quality of life, *M* mean, *SD* standard deviation

**Table 3:** Descriptive statistics for oral health-related knowledge, attitudes (N = 281)

	M ± SD (range)	N (%)		M ± SD (range)	N (%)
K1	0.61 ± 0.49(0–1)	172(61.2)	A1	0.92 ± 0.27(0–1)	259(92.2)
K2	0.65 ± 0.48(0–1)	183(65.1)	A2	0.63 ± 0.48(0–1)	178(63.3)
K3	0.47 ± 0.50(0–1)	132(47.0)	A3	0.60 ± 0.49(0–1)	169(60.1)
K4	0.47 ± 0.50(0–1)	133(47.3)	A4	0.88 ± 0.32(0–1)	248(88.3)
K5	0.60 ± 0.49(0–1)	169(60.1)	A5	0.91 ± 0.28(0–1)	257(91.5)
K6	0.10 ± 0.30(0–1)	27(9.6)			
K7	0.11 ± 0.32(0–1)	32(11.4)			
K8	0.79 ± 0.41(0–1)	222(79.0)			
K	3.81 ± 2.06(0–8)	47.6%	A	3.95 ± 1.31(0–5)	79.1%

*M* Mean, *SD* standard deviation, *K* total awareness rate of oral health knowledge, *A* total holding rate of positive attitudes toward oral health

Regarding oral health practices, less than half brushed their teeth twice a day or more (49.1%), fewer used electric toothbrushes (1.1%), floss (9.3%) and mouthwash (3.2%), and 34.9% were still accustomed to using toothpicks. Only 7.1% reported using fluoride toothpaste, 92.5% rinsed with tap water, and the majority replaced their toothbrush after more than 3 months (55.2%). The participants’ main oral cleaner after admission was themselves (96.4%). A total of 91.1% did not clean or need to clean their dentures, with 4.3% cleaning their dentures once a day. A total of 38.1% had never seen a dentist, 46.2% had not seen a dentist for more than a year, and the main reason for the last visit was discomfort from tooth, gum or mouth pain (43.8%). The main reasons for not having visited a dentist in the past year were no dental problems (56.2%) and no serious dental disease (20.6%). A total of 77.2% had never been to a clinic or hospital for dental cleaning, and 12.5% had their teeth cleaned more than once every three years.

### OHRQoL of hospitalised stroke patients

In this study, participants had a mean OHIP-14 score of 8.37 ± 6.67, with the highest mean score for the painful discomfort of the mouth dimension (3.11 ± 2.13). The most common negative effect on patients was pain (13.5%), followed by discomfort while eating (10%), whereas irritability and embarrassment showed no negative effects (Table 4).

**Table 4:** OHIP-14 scale entries that negatively impacted patients (N = 281)

Dimensions	Items	N (%)
Diminished independence	1 Affects pronunciation	5(1.8%)
	11 Easily lose temper with others	0
	12 Difficult to complete daily tasks	1(0.4%)
	13 Feeling that life is less satisfying	1(0.4%)
	14 Can't do anything	1(0.4%)
Psychological discomfort	5 Feeling uncomfortable in front of other people	5(1.8%)
	6 Feeling nervous and uneasy	1(0.4%)
	10 Having embarrassing moments	0
Discomfort in physical functioning	7 Dissatisfied with your diet	7(2.5%)
	8 Stopped in the middle of a meal	3(1.1%)
	9 Not being able to rest well	3(1.1%)
Pain and discomfort of the mouth	2Taste sensation becomes worse	4(1.4%)
	3 Experiencing significant pain	38(13.5%)
	4 Eating anything is uncomfortable	28(10%)

### Factors associated with OHRQoL

In univariate analysis, we found that participants’ OHRQoL was associated with age (*P* = 0.001), occupation (*P* = 0.007), monthly household income per capita (*P* = 0.027), use of dentures (*P* = 0.002), number of teeth (*P* < 0.000), number of missing teeth (*P* < 0.000), toothache (*P* < 0.000), absence of malnutrition (*P* = 0.018), self general health assessment (*P* = 0.015), self-rated oral health assessment (*P* < 0.000), denture cleaning method (*P* = 0.045), frequency of denture cleaning (*P* = 0.047), time since last dentist visit (*P* < 0.000), reason for last dentist visit (*P* < 0.000), and reason for not visiting a dentist in the past year (*P* < 0.000). However, when we used OHRQoL as the dependent variable, the significant variables were placed into a multiple linear regression equation, and stepwise regression analysis was used to screen the variables. Thus, the influencing factors in the model were age, toothache, self-rated oral health assessment, time since last dentist visit, and reason for not visiting the dentist in the past year (Tables 5 and 6).

**Table 5:** Spearman's correlation coefficient was used for OHRQoL correlation analysis(N = 281)

Variable	OHRQoL	*p*− value
OHRQoL	1	
Age	.197**	.001
Occupation	.162**	.007
Monthly household income per capita	− 132*	.027
With or without dentures	.183**	.002
Number of teeth	− .261**	< .000
Number of missing teeth	.267**	< .000
Toothache	.299**	< .000
Absence of oral malnutrition	− 141*	.018
Self general health assessment	− 146*	.015
Self− rated oral health assessment	− 365**	< .000
Denture cleaning method	.120*	.045
Frequency of denture cleaning	.119*	.047
Time since last dentist visit	.217**	0.001
Reason for last visit to the dentist	.219**	< .000
Reason for not having visited a dentist in the past year	.404**	< .000

OHRQoL: Oral health-related quality of life;* the mean of correlation for OHRQoL was significant (*p* < 0.05);** the mean of correlation for OHRQoL was significant (*p* < 0.01)

**Table 6:** Multiple linear regression was used for OHRQoL influence factor analysis* (N = 281)

	Standardization coefficients	t	*P*-value	95.0%CI of B	
	Beta			Lower	Upper
Constants		3.734	< .000	3.342	10.795
Self-rated oral health assessment	− 0.22	− 4.144	< .000	− 2.49	− 0.886
Toothache	0.242	4.64	< .000	2.716	6.718
Reason for not having visited a dentist in the past year	0.269	5.102	< .000	0.437	0.987
Age	0.144	2.686	0.008	0.344	2.232
Time since last dentist visit	0.113	2.094	0.037	0.021	0.687

*OHRQoL* Oral health-related quality of life

Stepwise regression model fit Adjusted R Squared was 0.257, *p* < 0.000

## Discussion

The purpose of this study was to assess the level of oral health KAP among stroke inpatients and to analyse the factors influencing OHRQoL among stroke inpatients. The results of this study showed that age, toothache, self-rated oral health status, time since last dentist visit, and reason for not visiting the dentist in the past year were factors that influenced the OHRQoL of stroke patients. To the best of our knowledge, there are few domestic studies on the factors influencing the OHRQoL of stroke patients, and this study is the first in China to report the level of oral health KAP among stroke patients.

Our results showed that the rate of oral health knowledge among stroke inpatients is not satisfactory. This is much lower than the results of the Fourth National Survey on the Oral Health of the Population in the Chinese mainland (60.1%) [24]. The rate of positive attitudes towards oral health was found to be satisfactory but still lower than the results of the Fourth National Survey on the Oral Health of the Population in the Chinese mainland (84.9%) [24]. In other studies, oral health literacy levels were lower among stroke patients than among middle-aged and older adults, residents and migrant workers [25–27]. The low knowledge levels of fluoride and fissure sealants among stroke patients in this study is consistent with findings from other studies in which older adults and residents were surveyed [28, 29]. Notably, we found that the levels of oral health knowledge and attitudes were not coordinated. Oral health knowledge was also shown to be poor compared to oral health attitudes and practices in the study by Wong et al. (2020) [30]. This may be attributed to the fact that people are increasingly concerned about their physical health as well as their quality of life, and participants had positive attitudes towards oral health, but their knowledge of oral health was limited due to their education level [31], monthly household income [32] and permanent home address [33]. Members of low-income or rural populations were more likely to have oral health knowledge [34]. In addition, in this study, the main way that participants accessed oral health knowledge was online. With the rapid development of the internet, the ability of society to share ideas and knowledge has increased exponentially, and an increasing number of people are accessing health information through this channel [35]. In this regard, Hanna et al. (2017) emphasized that online health services are a common way for patients to seek oral health-related information and can be used to improve oral health-related knowledge by providing patients with internet guidance [36].

In terms of oral health practices, we found that participants brushed their teeth more than twice a day more frequently than of the adults surveyed in the National Population Oral Health Survey (36.1%) [24]. This may be because the participants were surveyed in a tertiary hospital in a first-tier city in China, where the participants’ oral health behaviors were better than the national average. Only 7.1% of the participants reported using fluoride toothpaste, which may be because in this study, they were not sure whether the toothpaste they were using contained fluoride. Some studies have shown that better educated and younger participants were more likely to know about fluoride [31]. In this study, the majority of participants had no dentures (66.9%), andas among those who did, most cleaned their dentures once a day (4.3%), suggesting that participants did not pay much attention to the cleaning of their dentures. Regarding the patients’ dentist visit behaviour, we found that the frequency of visits was low. According to the reasons for the last dentist visit, the most important reason was dental, gum, or oral pain and discomfort, which is also consistent with the results of the OHRQoL scores in this study. Most of the participants did not visit the dentist for a year because they thought there were no dental problems (56.2%) or their dental disease was not serious (20.6%). Their health awareness about dental cleaning was not strong however, more than half of the participants had a smoking habit (57.3%). Rasouli-Ghahroudi et al. (2016) showed that patients with coronary heart disease had an overall moderate level of knowledge and attitudes, but their practices were below moderate [37]. A systematic review found that diabetic patients had a lack of oral health knowledge, poorer oral health attitudes, and fewer denist visits [38]. According to Andersen's model of health behaviour, individual’s attitudes and health knowledge gradually influence their health-seeking behaviour [39]. Therefore, effective oral health education should be provided to stroke inpatients to improve their oral health behaviours.

The OHIP was developed in the 1990s by Slade et al. and has produced different versions depending on the number of problems [40]. Today, the OHIP-14 is widely used for different research questions. It is therefore well suited for use in clinical studies and is a valid assessment tool [12]. Various studies have reported mean OHIP-14 total scores between 2.87 and 33 regarding the oral health profiles of stroke patients [41–45]. Studies have shown that patients’ ADL levels, stroke disability, and recovery time affect OHRQoL [41, 43, 45]. In the present study, stroke inpatients were selected as participants, but most of them were mildly dependent or fully independent (43.8% and 42.3%, respectively), and more than half of them had their first stroke within 1 month (58.4%), so their oral health was at a better level relative to other studies, and no correlation was found with OHRQoL. Although the OHIP initially defined seven domains [46], recent studies as well as the Chinese version of the scale have focused on four dimensions of OHRQoL. We differed from other studies in our results due to differences in the division of dimensions and in the definition of the impact of producing an OHRQoL [46].

A systematic review showed that the quality of oral health is poorer among women [47]. In contrast, the majority of patients in this study were male. In the present study, similar to other studies [43], the older the stroke patients were, the worse the OHRQoL. The lower the socioeconomic status of the individual was, the worse their OHRQoL [48]. However, in the correlation analysis, unemployed patients and those with lower per capita household incomes had poorer OHRQoL, but there were no confounding associations in the regression analyses. The majority of patients in this study were married and had a family member as the primary caregiver. In contrast, the oral health status of married couples was correlated with oral health behaviours, with the oral health status of husbands being directly related to their oral health behaviours and smoking habits, whereas the oral health status of wives was only directly related to their oral health behaviours [49]. This study showed a significant correlation between swallowing disorders and OHRQoL [50–52]. Malnutrition may contribute to the development of oral diseases, reduce resistance to oral bacteria, and affect oral health [53]. However, in the present study, we did not find a significant correlation between swallowing function and nutritional status or OHRQoL. This may be because only 1.2% of patients with swallowing disorders in this study had swallowing function above grade III and only 0.4% of patients were at nutritional risk. Additionally, there was no significant correlation between stroke-related factors such as the number of stroke occurrences, type of stroke, and stroke duration and OHRQoL in this study. Although the current evidence is insufficient, some studies found that the degree of stroke disability and upper limb motor function may be associated with OHRQoL [41, 43, 45, 54].

Stroke patients have a substantially lower OHRQoL, a larger number of missing teeth, and poorer plaque and gingival index scores than nonstroke patients [55, 56]. In addition, stroke patients tend to have a higher burden of dental caries, periodontitis, and tooth loss, as well as a lower frequency of dental visits [55, 56]. Oral diseases, including tooth loss, can negatively affect OHRQoL [57, 58]. This is also consistent with our findings. Moreover, in the correlation analysis of OHRQoL as a subaspect of overall health-related quality of life [57, 58], we found that patients with a better self-rated general health status had better OHRQoL. However, in the regression analysis, we did not find a significant confounding correlation. In addition, patients’ denture cleaning methods, denture cleaning frequency, time since last dentist visit, and OHRQoL were correlated. The results showed that patients with less frequent denture cleaning and a longer time since their last dentist visit had worse OHRQoL, but we still did not find a significant confounding correlation in the regression analysis. Finally, by regression analysis, we found that age, toothache, evaluation of dental and oral status, reason for not having visited the dentist in the past year, and time since the last visit to the dentist were factors influencing OHRQoL among patients hospitalised with a stroke in this study. This suggests to us that health care professionals should support oral hygiene and dental visits and promote oral health education for patients to improve their oral health and OHRQoL [59].

## Limitations

This study has several limitations. First, the assessment of oral diseases, such as periodontitis, was not considered. Previous studies have suggested that periodontal disease may be associated with stroke onset [7, 60]. Second, the participants in this study came from three hospitals in Guangdong Province, two of which specialize in cerebrovascular diseases. However, no other regional general hospitals were included, so it may be inappropriate to generalize the results to a broader Chinese population. Third, the questionnaire was based on patient self-report and was administered by interviewers, which may be subject to recall and social desirability bias. Fourth, because the effect of stroke-related factors did not appear significant in this study, a longitudinal study was considered to determine the effect of stroke course on OHRQoL or to add stroke factors such as stroke disability and cognitive impairment for assessment to target oral health guidance for patients with different levels of stroke disease.

## Conclusion

In conclusion, the OHRQoL of patients hospitalised with stroke is moderate, and their oral conditions still need to be improved. The OHRQoL of patients is influenced by patients' age, dental pain, self-rated oral health assessments and oral health behaviours. Therefore, in clinical work, attention to elderly stroke patients and patients with poor oral status and poor oral health behaviours should be strengthened, swallowing function, nutritional function and self-care ability of patients should be assessed in a timely manner, and oral health interventions and guidance should be given in an early and targeted manner.

## Data Availability

The dataset generated or analysed during the current study is not publicly available as it is part of the first author's master's degree project for this study but will be available upon completion of the other sub-studies. Available from the corresponding author upon reasonable request.
